# Fatty Acid Amide Hydrolase Signaling and Ovarian Disorders: From Molecular Mechanism to Clinical Significance

**DOI:** 10.3390/molecules31030556

**Published:** 2026-02-05

**Authors:** Qinghe Lin, Zhenghong Zhang, Defan Wang, Fan Wang, Zhengchao Wang

**Affiliations:** 1 Provincial Key Laboratory for Developmental Biology and Neurosciences, College of Life Sciences, Fujian Normal University, Fuzhou 350007, China; qinghelin2025@163.com (Q.L.); zhangzh@fjnu.edu.cn (Z.Z.); 2 Provincial University Key Laboratory of Sport and Health Science, Fujian Normal University, Fuzhou 350007, China; 3 Fujian Provincial Key Laboratory of Reproductive Health Research, School of Medicine, Xiamen University, Xiamen 361102, China; defanwang@126.com

**Keywords:** fatty acid amide hydrolase, ovarian function, endocannabinoid system, follicular development, reproductive diseases

## Abstract

Fatty acid amide hydrolase (FAAH) is a central component of the endocannabinoid system (ECS), where it primarily regulates intracellular levels of anandamide (AEA) through enzymatic hydrolysis. Although FAAH has been extensively studied in neural and immune contexts, its involvement in female reproductive physiology is receiving increasing attention. Accumulating evidence indicates that FAAH participates in several important ovarian processes, including follicular development, steroid hormone synthesis, ovulation, and luteal function. In this review, we outline the biochemical properties of FAAH and its spatial distribution in ovarian tissues, with a particular focus on how FAAH-mediated AEA metabolism contributes to intraovarian signaling. Furthermore, we highlight the potential implications of altered FAAH activity in ovarian disorders such as polycystic ovary syndrome (PCOS), premature ovarian insufficiency (POI), and infertility. By integrating molecular observations with clinical findings, this work provides updated perspectives on FAAH as both a physiological regulator and a potential therapeutic target in reproductive medicine.

## 1. Introduction

Fatty acid amide hydrolase (FAAH) is a key metabolic enzyme in the endocannabinoid system (ECS), responsible for hydrolyzing anandamide (AEA) and other fatty acid amides [1,2,3,4]. By controlling local AEA concentrations, FAAH modulates intracellular signaling and maintains the balance of the ovarian microenvironment [1,5]. Increasing evidence indicates that FAAH plays a central role in ovarian physiology, influencing granulosa cell function, oocyte maturation, steroidogenesis, ovulation, and luteal maintenance [4,5,6,7,8,9,10,11,12,13,14,15,16,17].

Within the ovary, FAAH is predominantly expressed in granulosa cells, corpus luteum cells, and the ovarian surface epithelium (OSE), with lower levels in oocytes [6,9]. Its expression is also detectable in subepithelial cords and the tunica albuginea, suggesting a potential role in coordinating follicular development and ovarian structural integrity [4,5,6,7]. This spatial distribution implies that FAAH regulates both endocrine and paracrine signaling pathways within the ovary.

The ECS, consisting of endogenous ligands (e.g., AEA), cannabinoid receptors (CB1 and CB2), and metabolic enzymes (FAAH), is widely expressed in reproductive tissues [4,8,9,10,11,12,13,14,15,16,17]. In the ovary, FAAH co-localizes with CB2 receptors in granulosa and luteal cells, suggesting that AEA degradation can indirectly modulate receptor activity and downstream signaling [4,5,6,7,8,10,11]. By controlling AEA availability, FAAH influences steroidogenic enzyme expression, granulosa cell survival, and oocyte competence, highlighting its integrative role in the ovarian microenvironment [1,7,12,13,14,15,16,17].

Disruption of FAAH activity leads to AEA levels being altered, which can impair follicular growth, ovulation, and luteal function, thereby contributing to reproductive disorders such as polycystic ovary syndrome (PCOS), premature ovarian insufficiency (POI), and infertility. This review focuses on the molecular characteristics, ovarian expression patterns, regulatory mechanisms, and functional implications of FAAH, emphasizing its role in modulating the ovarian microenvironment and its potential as a therapeutic target.

Additionally, a comprehensive literature search was conducted using PubMed/Medline, Web of Science Core Collection, and Scopus. Search terms included “fatty acid amide hydrolase” or “FAAH” and “ovary”, “ovarian”, “granulosa cells”, “luteal cells”, “folliculogenesis”, “ovulation”, “endocannabinoid system”, or “anandamide”. Only English-language articles published up to September 2025 were considered. After duplicate removal, titles/abstracts were screened, followed by full-text evaluation. Studies were included if they directly and indirectly addressed FAAH expression, regulation, or function in ovarian or reproductive contexts.

## 2. FAAH Structure and Function

**Figure 1:**
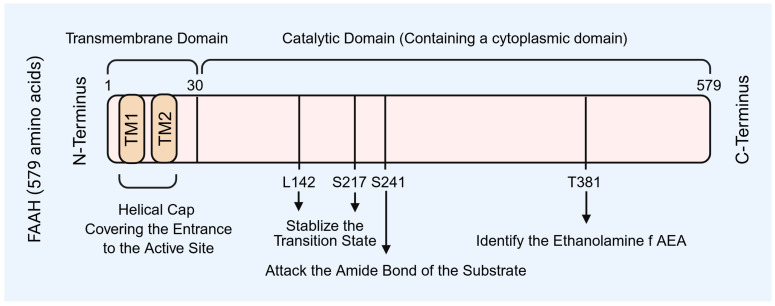
Fatty acid amide hydrolase (FAAH) structure and function. FAAH enzyme includes three principal regions: an N-terminal transmembrane domain, a central catalytic core, and a C-terminal cytoplasmic domain. FAAH: fatty acid amide hydrolase; AEA: anandamide. TM: transmembrane domain. Created by Bio-Render.

FAAH belongs to the serine hydrolase family and is primarily anchored to the endoplasmic reticulum (ER) membrane. Its molecular architecture underlies its catalytic efficiency, substrate specificity, and regulatory potential within ovarian cells. The enzyme consists of three principal regions: an N-terminal transmembrane domain, a central catalytic core, and a C-terminal cytoplasmic domain (Figure 1).

### 2.1. Transmembrane Domain (N-Terminal 1–30 aa)

The N-terminal domain contains two α-helices embedded in the ER membrane, forming a helical cap that partially covers the entrance to the catalytic site (Figure 1) [6,18,19,20]. This arrangement positions FAAH near lipid substrates such as AEA, facilitates conformational changes that regulate substrate accessibility, and mediates interactions with small molecule modulators, including selective inhibitors like OL-135 [6,18]. In ovarian cells, this localization enables FAAH to efficiently modulate local AEA concentrations, maintaining the microenvironment required for granulosa cell survival and oocyte maturation.

### 2.2. Catalytic Core (31–579 aa)

The catalytic domain contains a triad of residues—Ser241, Ser217, and Lys142—coordinated with Tyr381—that execute nucleophilic attack on the amide bond of AEA (Figure 1) [20,21,22,23]. This structure accounts for FAAH’s high substrate specificity and catalytic efficiency. By hydrolyzing AEA, FAAH directly regulates endocannabinoid signaling within granulosa cells, luteal cells, and the ovarian stroma, thereby influencing steroidogenesis, follicular development, and luteal maintenance.

### 2.3. C-Terminal Cytoplasmic Domain

The function of the C-terminal domain remains incompletely defined but may contribute to protein stability, intracellular trafficking, or interactions with other signaling proteins (Figure 1). Its presence could influence FAAH’s spatial distribution within the ovary and facilitate dynamic responses to hormonal or paracrine signals [19,20,21,22,23].

### 2.4. Functional Implications

FAAH preferentially hydrolyzes AEA but can also act on other fatty acid amides, reflecting its broad role in regulating endocannabinoid tone (Figure 1) [6,22,24]. In the ovary, this enzymatic activity ensures that local AEA concentrations remain within ranges compatible with granulosa cell proliferation, oocyte maturation, luteal hormone synthesis, and tissue remodeling. Consequently, any structural or regulatory perturbation that diminishes FAAH activity can disrupt ovarian microenvironment homeostasis and impair reproductive competence.

## 3. FAAH Expression and Regulation in the Ovary

**Figure 2:**
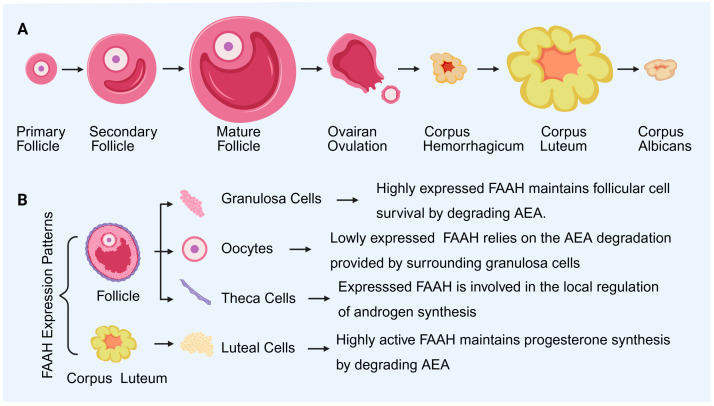
FAAH expression and cellular localization during follicle development in the ovary. (**A**): Pattern diagram of follicle development and luteal formation. (**B**): The expression patterns and cellular localization of FAAH. FAAH: fatty acid amide hydrolase; AEA: anandamide. Created by Bio-Render.

FAAH expression in the ovary is cell-type specific and dynamically regulated, reflecting its central role in controlling local AEA concentrations and maintaining ovarian homeostasis (Figure 2). Its distribution and regulation are tightly linked to granulosa cell function, oocyte maturation, luteal activity, and overall reproductive competence.

### 3.1. Cellular Localization

FAAH is highly expressed in granulosa cells of dominant follicles, where it maintains low AEA levels to support cell survival and steroidogenesis [4]. In contrast, granulosa cells of atretic follicles exhibit reduced FAAH expression, leading to AEA accumulation and activation of CB1-mediated apoptotic pathways, thereby promoting follicular regression [4,16].

In luteal cells, FAAH expression rises during luteinization, facilitating progesterone synthesis by controlling AEA concentrations. During luteal regression, FAAH levels decline, allowing AEA to trigger luteal cell apoptosis, consistent with its role in cyclic ovarian remodeling [4,25].

Oocytes express minimal FAAH; their AEA environment is largely regulated by surrounding granulosa cells. Experimental inhibition of FAAH during in vitro oocyte maturation increases AEA levels and impairs oocyte quality, indicating the enzyme’s role in oocyte competence [5,11,12,13,26].

FAAH expression in theca cells is lower than in granulosa cells but contributes to local androgen regulation, particularly under conditions such as PCOS [9,13].

Together, these patterns suggest that FAAH does not have a uniform role in the ovary but is embedded in cell-specific regulatory circuits that coordinate follicular development and luteal dynamics.

### 3.2. Hormonal Regulation

**Figure 3:**
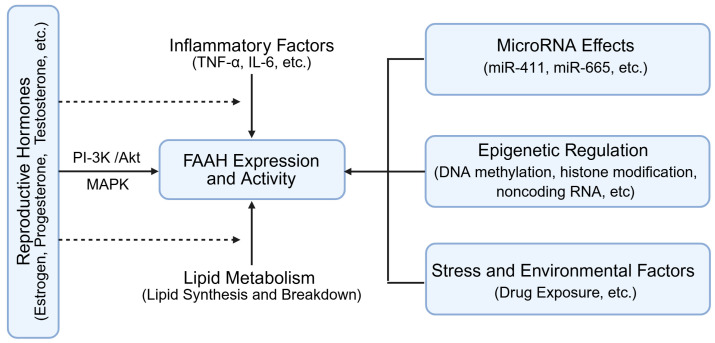
FAAH expression and activity through hormonal, epigenetic and microRNA regulation. FAAH: fatty acid amide hydrolase. TNF-α: tumor necrosis factor-α; IL-6: interleukin-6; PI-3K: phosphatidylinositol 3-kinase; AKT/PKB: protein Kinase B; MAPK: mitogen-activated protein kinase. Created by Bio-Render.

Reproductive hormones modulate FAAH expression in the ovary, integrating systemic endocrine signals with local ECS activity (Figure 3). Estrogen, progesterone, and testosterone influence FAAH transcription and enzymatic activity via receptor-mediated pathways [17,27,28,29].

Estrogen can indirectly downregulate FAAH by suppressing inflammatory cytokines such as tumor necrosis factor-α (TNF-α) and interleukin-6 (IL-6), which themselves enhance FAAH expression [28,30]. Conversely, estrogen may also upregulate FAAH through the activation of downstream phosphatidylinositol 3-kinase (PI-3K)/protein Kinase B (AKT/PKB) or mitogen-activated protein kinase (MAPK) pathways, reflecting context-dependent regulatory effects [31,32,33,34,35,36,37].

Progesterone and testosterone similarly impact FAAH expression, linking steroid hormone feedback to the modulation of AEA levels. This hormonal regulation ensures that AEA concentrations fluctuate appropriately across the follicular and luteal phases, maintaining ovarian microenvironment homeostasis.

### 3.3. Epigenetic and MicroRNA Regulation

FAAH expression is also fine-tuned by microRNAs and epigenetic modifications (Figure 3). For example, miR-411 and miR-665 bind FAAH mRNA, reducing its translation and promoting degradation. Histone acetylation (e.g., H3K9ac) and DNA methylation further influence transcriptional activity, allowing environmental and developmental cues to shape FAAH expression patterns. Although most of this evidence derives from neural or inflammatory models, these mechanisms may also be relevant to ovarian FAAH regulation [30,38,39,40,41,42,43].

External factors, including stress and xenobiotic exposure, can alter FAAH expression through epigenetic mechanisms (Figure 3). For example, alcohol or chronic stress has been shown to modify DNA methylation or microRNA profiles affecting FAAH. Most of this evidence derives from cortex, astrocyte and other models, but the molecular mechanisms of external factors may also impact the ovarian AEA milieu [41,42,43,44].

Based on the above regulatory mechanisms of FAAH, we can find that dynamic regulation of FAAH ensures that AEA levels remain compatible with follicular growth, granulosa cell survival, oocyte maturation, and luteal function. Hormonal fluctuations, microRNA activity, and environmental inputs collectively modulate FAAH expression, integrating systemic and local signals to preserve ovarian microenvironment homeostasis.

## 4. Molecular Mechanisms of FAAH Regulating Ovarian Functions

FAAH modulates ovarian physiology primarily by controlling intraovarian AEA concentrations. Through this mechanism, it influences follicular development, steroid hormone synthesis, ovulation, luteal function, and ovarian aging. To improve clarity and avoid cross-species overinterpretation, the key findings related to FAAH signaling in ovarian tissues across different species are summarized in Table 1.

### 4.1. Regulation of Follicle Development

**Figure 4:**
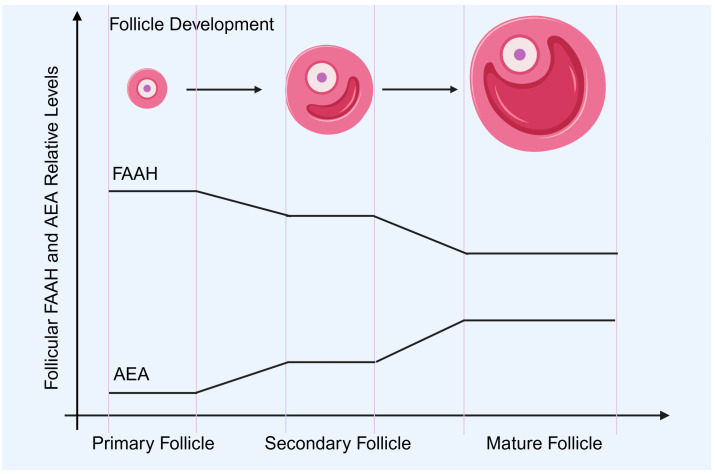
The changes in FAAH and AEA expression levels during follicle development. This schematic diagram mainly shows the expression changes in FAAH and AEA during ovarian follicle development, indicating the regulatory effect of FAAH on AEA. There are no specific expression changes during ovarian ovulation and subsequent corpus luteum formation shown here. FAAH: fatty acid amide hydrolase; AEA: anandamide. Created by Bio-Render.

FAAH expression changes dynamically during follicular growth, ensuring that AEA concentrations remain within a physiologically permissive range for follicular development and oocyte maturation (Figure 4) [8,10,50]. In growing secondary and antral follicles, granulosa cells exhibit relatively high FAAH activity, which maintains AEA at a low baseline level. This environment supports granulosa cell proliferation, inhibits premature apoptosis, and favors the establishment of a stable intrafollicular microenvironment [16,51]. The reduction in FAAH expression observed in atretic follicles coincides with a marked elevation of AEA, which activates CB1-dependent apoptotic pathways, thereby promoting follicular atresia [4,10]. These findings indicate that fluctuations in FAAH activity are not merely correlative but play a determining role in follicle selection and survival.

The relationship between FAAH and oocyte maturation is also closely regulated (Figure 4). Follicles that progress to preovulatory stages show increased FAAH expression in mural granulosa and cumulus cells, resulting in decreased AEA levels within the follicular fluid [8,44]. This low-AEA state has been associated with improved oocyte metabolic stability, normal meiotic spindle formation, and enhanced developmental competence. In contrast, pharmacological inhibition of FAAH during in vitro maturation elevates intrafollicular AEA and disrupts cumulus–oocyte communication, often accompanied by impaired mitochondrial function and reduced fertilization potential [8,16,51].

Beyond cell survival and maturation, FAAH appears to participate in granulosa–oocyte paracrine signaling. Growth factors such as growth differentiation factor 9 (GDF9) and bone morphogenetic protein 15 (BMP15), released by the oocyte, may influence FAAH expression in adjacent granulosa cells, suggesting the existence of a bidirectional regulatory loop [52,53,54,55,56,57]. This coordinated signaling ensures that AEA fluctuations mirror follicular developmental needs, linking metabolic enzyme regulation with reproductive cell fate decisions. Taken together, FAAH acts as a local molecular gatekeeper that shapes the trajectory of follicular growth, atresia, and oocyte quality.

### 4.2. Regulation of Steroid Hormone Synthesis

**Figure 5:**
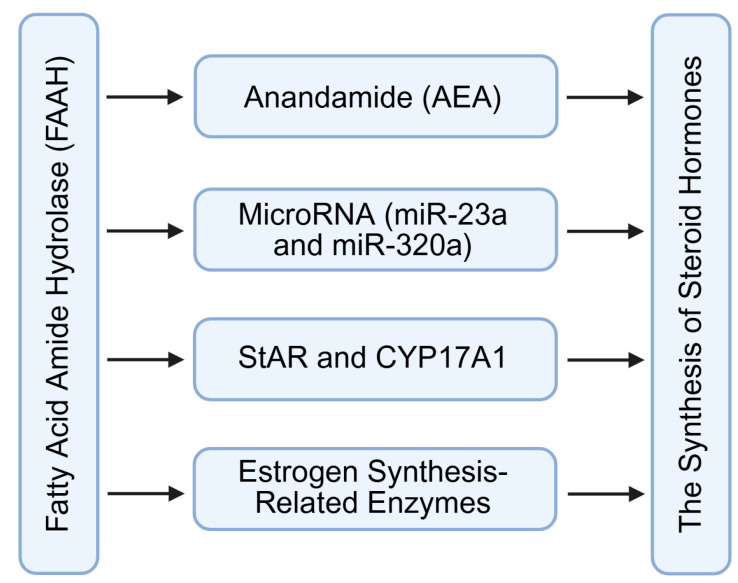
Regulation of steroid hormone synthesis by FAAH signaling through controlling intracellular AEA levels or microRNAs. FAAH: fatty acid amide hydrolase; AEA: anandamide; StAR: steroidogenic acute regulatory protein; CYP17A1: cytochrome P450 family 17 subfamily A member 1. Created by Bio-Render.

FAAH regulates ovarian steroidogenesis mainly by controlling intracellular AEA levels in granulosa and luteal cells (Figure 5) [58,59,60]. In growing follicles, FAAH expression tends to increase with granulosa cell differentiation, which contributes to maintaining relatively low AEA concentrations in the follicular microenvironment. This reduction in AEA relieves its inhibitory effect on steroidogenic gene transcription. In particular, adequate FAAH activity supports the expression of aromatase cytochrome p450 (CYP19), thereby promoting the conversion of androgens to estradiol during follicular maturation [14]. When FAAH activity is reduced, AEA accumulation can inhibit aromatase function through CB1-linked signaling pathways, ultimately affecting estrogen biosynthesis and impairing follicular development [58,59,60].

In luteal cells, FAAH plays an analogous role in maintaining progesterone production (Figure 5). By degrading excess AEA, FAAH prevents CB1-mediated suppression of steroidogenic acute regulatory protein (StAR) and 3β-hydroxysteroid dehydrogenase, two key enzymes required for cholesterol transport and progesterone synthesis [15,51]. Hence, FAAH contributes to luteal stability and the maintenance of the post-ovulatory endocrine environment.

In addition to direct effects on steroidogenic enzymes, AEA signaling intersects with post-transcriptional regulatory mechanisms (Figure 4). Recent studies indicate that AEA may modulate the expression or activity of microRNAs such as miR-23a and miR-320a, which in turn target transcripts encoding steroidogenic regulators [4,14,59]. Through these interactions, FAAH indirectly influences gene networks governing hormone production, linking metabolic enzyme activity with fine-tuned transcriptional and post-transcriptional regulatory circuits.

Overall, FAAH activity ensures that estrogen and progesterone synthesis proceed in a manner synchronized with follicular growth, ovulation, and luteal maintenance. Disruptions in FAAH-mediated AEA regulation may therefore contribute to endocrine imbalance and ovarian dysfunction.

### 4.3. Regulation of Ovulation and Luteal Function

**Figure 6:**
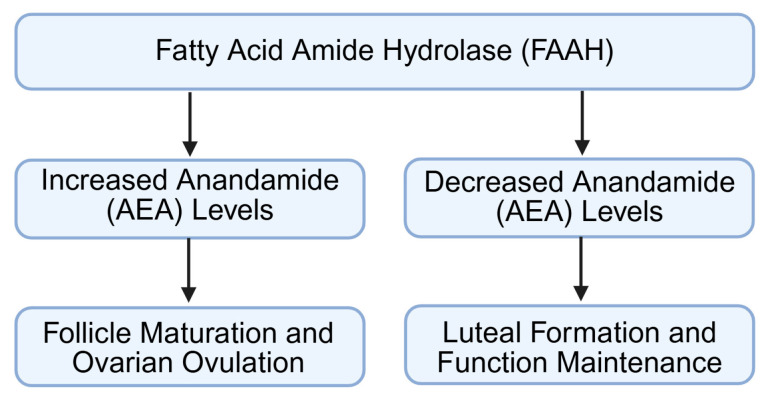
Expression changes in and regulation of FAAH and AEA signaling during ovulation and luteal formation. FAAH: fatty acid amide hydrolase; AEA: anandamide. Created by Bio-Render.

FAAH plays an important regulatory role during ovulation by modulating AEA-mediated signaling within the periovulatory follicle (Figure 6) [8,10,16,61]. As the pre-ovulatory LH surge initiates a shift in granulosa cell function, FAAH expression is increased to reduce AEA levels. This reduction is necessary to prevent excessive CB1 receptor activation, which would otherwise inhibit the transcription of ovulatory mediators such as Ptgs2 (cyclooxygenase-2) and Pappa (pregnancy-associated plasma protein-A) [8,61,62,63]. Adequate FAAH activity, therefore, supports the biochemical cascade required for follicle wall remodeling, cumulus expansion, and oocyte release. When FAAH levels are insufficient, elevated AEA can disrupt these signaling events, potentially leading to delayed or incomplete ovulation.

Following ovulation, FAAH continues to influence reproductive function by regulating luteal formation and maintenance [4,11,15,64]. In the newly formed corpus luteum, FAAH helps maintain low AEA concentrations, promoting luteal cell survival and progesterone production. This protective effect is achieved by limiting CB1-driven pro-apoptotic pathways, thereby ensuring the functional stability of the luteal phase (Figure 6) [15]. Over the course of the cycle, a natural decline in FAAH activity contributes to increased AEA levels, which participate in the initiation of luteal regression by promoting apoptosis and reducing steroidogenic capacity [16,63].

This tightly controlled temporal adjustment in FAAH expression, high immediately after ovulation and lower during luteolysis, demonstrates how FAAH coordinates the transition from follicular rupture to corpus luteum maintenance and eventual regression. In this way, FAAH integrates ovulation dynamics with hormonal rhythmicity and the broader ovarian microenvironment.

### 4.4. Regulation of Ovarian Aging and Functional Decline

**Figure 7:**
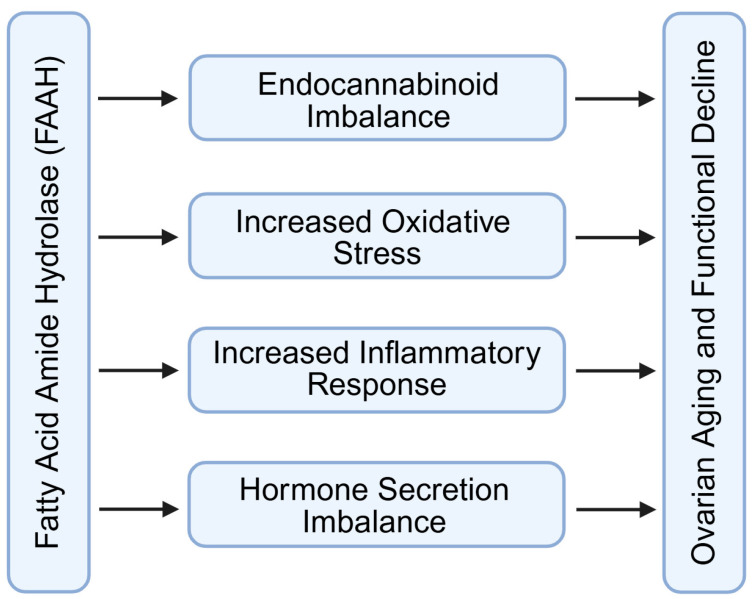
Regulation of ovarian aging and functional decline by FAAH signaling through endocannabinoid/hormone imbalance and increased oxidation/inflammation. FAAH: fatty acid amide hydrolase. Created by Bio-Render.

Ovarian aging is characterized by a progressive reduction in follicle number, a decline in oocyte developmental potential, and alterations in steroid hormone secretion patterns (Figure 7) [65,66,67,68,69]. Several studies indicate that FAAH expression gradually decreases with age in ovarian tissue, particularly within granulosa and luteal cells [4,5,12,13]. The reduction in FAAH activity leads to a relative accumulation of AEA in the ovarian microenvironment. Elevated AEA levels are associated with increased production of reactive oxygen species and impairment of mitochondrial function, both of which accelerate granulosa cell senescence and compromise oocyte metabolic homeostasis [3,13,16]. In addition, persistent AEA elevation can activate pro-inflammatory signaling pathways, further disrupting follicular support mechanisms and contributing to stromal fibrosis [67,68,69,70].

The influence of FAAH on ovarian aging is not limited to local tissue effects (Figure 7). Excess AEA has been shown to modulate hypothalamic activity and can suppress pulsatile gonadotropin-releasing hormone (GnRH) release [59,71,72,73]. Reduced GnRH availability affects pituitary secretion of FSH and LH, thereby creating a feedback loop that further diminishes follicular recruitment and steroidogenesis [71,72,73]. This endocrine adjustment may amplify age-related reproductive decline.

By controlling intracellular AEA concentrations (Figure 7), FAAH helps maintain redox balance, mitochondrial integrity, and anti-apoptotic signaling within follicular cells [8,16,74]. Adequate FAAH activity supports granulosa cell metabolic cooperation with the oocyte, preserves mitochondrial membrane potential, and limits inflammatory cytokine accumulation [75,76,77]. These protective actions collectively sustain oocyte quality and delay functional deterioration of the ovary [4,5,8,16,74,75,76,77]. Thus, FAAH can be regarded as a molecular checkpoint that stabilizes the ovarian microenvironment and modulates the pace of reproductive aging.

## 5. Clinical Significance of FAAH in Ovarian Disorders

**Figure 8:**
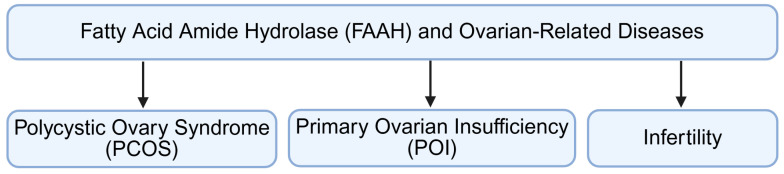
Clinical significance of FAAH in ovarian disorders. Aberrant FAAH may disrupt AEA homeostasis, impair ovarian functions, and then lead to ovarian disorders, including PCOS, POI, and infertility. FAAH: fatty acid amide hydrolase; PCOS: polycystic ovary syndrome; POI: premature ovarian insufficiency. Created by Bio-Render.

Aberrant FAAH expression or activity disrupts AEA homeostasis in the ovary, impairing follicular development, steroidogenesis, ovulation, and luteal function. Such dysregulation is associated with common reproductive disorders, including polycystic ovary syndrome, premature ovarian insufficiency, and infertility (Figure 8).

### 5.1. Polycystic Ovary Syndrome

**Figure 9:**
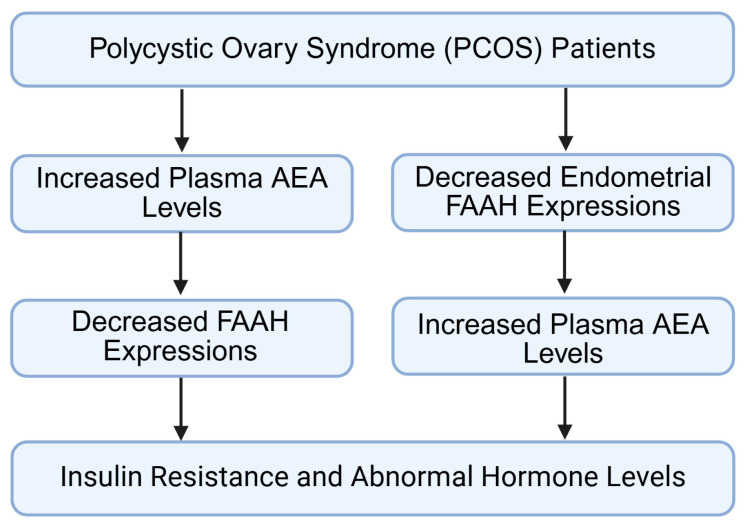
Role of fatty acid amide hydrolase in polycystic ovary syndrome. Impaired FAAH not only affects ovulation, but also alters the ovarian microenvironment, influencing insulin resistance and metabolic dysregulation. FAAH: fatty acid amide hydrolase; AEA: anandamide; PCOS: polycystic ovary syndrome. Created by Bio-Render.

In women with PCOS, FAAH expression is lower in the endometrium and in ovarian granulosa cells, which leads to an accumulation of AEA both locally within the follicular environment and systemically in circulation (Figure 9) [12,13]. Increased AEA disrupts several granulosa cell functions, including mitotic activity, responsiveness to FSH, and the expression of key steroidogenic enzymes such as aromatase [12,13]. These alterations impair estradiol synthesis and hinder the acquisition of oocyte developmental competence.

Under physiological conditions, FAAH levels fluctuate across the menstrual cycle, with higher expression during the peri-ovulatory and luteal phases (Figure 9) [4,11,15]. In PCOS, however, this cyclic variation is reduced, indicating that the endocannabinoid system fails to appropriately adjust to hormonal cues during follicle maturation [13].

The effects of elevated AEA extend beyond follicular development (Figure 9) [75,77,78,79]. High AEA concentrations can modify paracrine communication between granulosa cells and theca cells, favoring the upregulation of androgen-producing enzymes and contributing to the hyperandrogenic environment characteristic of PCOS [75,77,78,79,80].

Additionally, AEA may influence insulin signaling pathways within ovarian tissue, reinforcing insulin resistance and amplifying metabolic dysregulation (Figure 9) [13,77,78,79,80,81,82]. These combined effects suggest that impaired FAAH activity in PCOS does not merely affect ovulatory function, but also alters the structural and biochemical features of the ovarian microenvironment, influencing both reproductive and metabolic dimensions of the disorder [12,25,81,82,83].

### 5.2. Premature Ovarian Insufficiency

**Figure 10:**
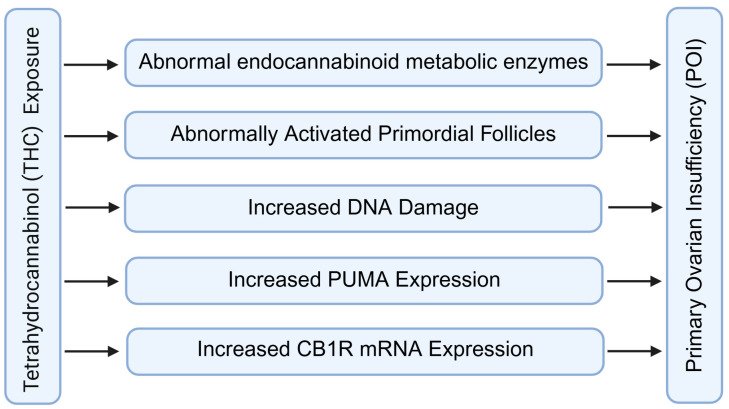
Role of fatty acid amide hydrolase in premature ovarian insufficiency. This section is mainly confirmed through THC toxicity experiments. In addition, some of the content about the effect of FAAH on POI is mainly inferred from the role of FAAH in other tissues and organs. FAAH: fatty acid amide hydrolase; POI: premature ovarian insufficiency; THC: tetrahydrocannabinol. Created by Bio-Render.

In premature ovarian insufficiency (POI), reduced FAAH activity impairs the clearance of AEA in granulosa and interstitial cells, resulting in sustained elevation of endocannabinoid tone within the ovarian microenvironment (Figure 10) [28,29]. Elevated AEA activates CB1-dependent signaling pathways that promote mitochondrial dysfunction and trigger caspase-mediated apoptosis, leading to premature loss of growing follicles and acceleration of ovarian reserve depletion [28,29,51].

Evidence from animal studies shows that cannabinoid exposure during adolescence disturbs the maturation of the endocannabinoid system and leads to persistent downregulation of FAAH expression in adulthood [16,51]. This disruption enhances sensitivity of primordial and early antral follicles to apoptotic cues, contributing to long-term ovarian insufficiency [28,51].

FAAH plays a key protective role by stabilizing AEA concentrations at levels compatible with granulosa cell survival and normal follicular metabolism [8,16]. Adequate FAAH activity helps limit oxidative stress, preserves mitochondrial integrity, and reduces inflammatory cytokine accumulation within the ovary [51,84,85,86].

When FAAH expression declines, the ovary becomes more vulnerable to additional insults, such as environmental toxins, metabolic imbalance, or chronic inflammation, thereby accelerating reproductive aging and functional decline [4,5,11,16]. Thus, FAAH acts as a buffering regulator that helps maintain follicular viability and slows the progression toward POI.

### 5.3. Infertility

**Figure 11:**
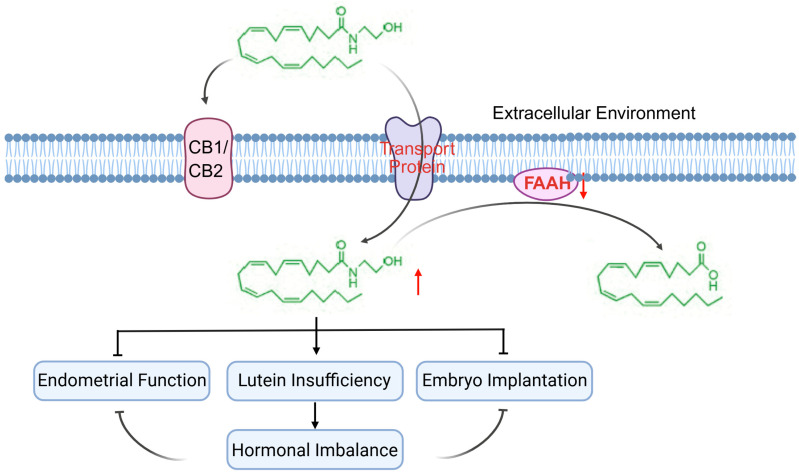
Role of fatty acid amide hydrolase (FAAH) in infertility. This graphical representation just presents the relationship between FAAH and AEA, and also indicates the role of FAAH in the context of broader AEA metabolic and signaling networks, while maintaining focus and avoiding speculative overextension. Red upward arrow (↑): Increase; Red downward arrow (↓): Decrease; FAAH: fatty acid amide hydrolase; AEA: anandamide. Created by Bio-Render.

The contribution of FAAH signaling to infertility about the ovary-centric mechanisms, including follicular development, oocyte competence, ovulatory dysfunction, luteal insufficiency and progesterone production, which has been thoroughly described in Section 4. In addition, FAAH also contributes to the functional integrity of the corpus luteum [4,15,16,45,46,47,48,49,87,88]. Diminished FAAH activity can lower progesterone synthesis by altering luteal cell survival signaling and steroidogenic enzyme expression, thereby weakening hormonal support during the peri-implantation window [15,22,62]. Inadequate progesterone compromises endometrial transformation and early pregnancy maintenance (Figure 11).

Furthermore, the extra-ovarian roles of FAAH in the female reproductive tract may be related to endometrial receptivity, decidualization, oviductal transport, and ectopic pregnancy. For example, FAAH is implicated in establishing endometrial receptivity and ensuring successful embryo implantation [45,46,47,48,49,88,89,90]. When FAAH expression is reduced, AEA concentrations rise within the endometrial microenvironment, which suppresses the production of leukemia inhibitory factor (LIF), a cytokine critical for embryo adhesion and decidualization [89]. Impaired LIF expression leads to inadequate preparation of the endometrial surface for blastocyst attachment. In addition, elevated AEA disrupts the coordinated crosstalk between epithelial and stromal cells, further weakening uterine receptivity [45,46,47,48,49,88,89,90]. While in the oviduct, excessive AEA interferes with ciliary movement and smooth muscle contraction, delaying embryo transport and increasing the likelihood of tubal retention [91]. This mechanism is consistent with clinical observations linking reduced FAAH activity and high circulating AEA levels to a higher risk of ectopic pregnancy [64,90,91,92]. Thus, endocannabinoid imbalance may affect not only intrauterine implantation but also early embryo trafficking.

Taken together, these findings indicate that FAAH functions at multiple reproductive checkpoints, aligning local microenvironmental conditions with systemic endocrine requirements, and thereby serving as a critical determinant of normal fertility (Figure 11). Moreover, to improve the clarity and avoid cross-species overinterpretation, FAAH and endocannabinoid signaling in human ovarian physiology and disorders are summarized in Table 2.

## 6. Potential Therapeutic Strategies Targeting FAAH

**Figure 12:**
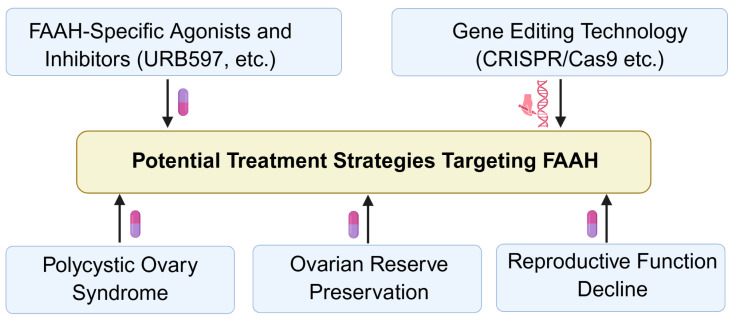
Potential therapeutic strategies targeting FAAH in ovarian disorders. Considering that the role of FAAH in ovarian physiology and pathology still needs further in-depth research, this section mainly focuses on the expression and regulation of FAAH in various tissues and proposes potential targeted FAAH therapy for ovarian diseases. FAAH: fatty acid amide hydrolase. Created by Bio-Render.

Given its central role in regulating intraovarian AEA levels, FAAH represents a promising therapeutic target for modulating ovarian microenvironment homeostasis. Strategies that restore or fine-tune FAAH activity could improve follicular development, steroidogenesis, ovulation, luteal function, and overall reproductive outcomes (Figure 12).

Selective FAAH inhibitors, such as URB597, have been explored in preclinical models to transiently elevate AEA levels under controlled conditions (Figure 12) [22,93,94,95]. Given the lack of current clinical evidence supporting FAAH inhibitors in ovarian disorders and the need for biomarker-guided stratification and tissue-selective delivery strategies, FAAH inhibition should increase AEA, and then the local ovarian modulation may improve follicular microenvironment homeostasis, particularly in contexts of suboptimal AEA degradation [12,13,14,16]. Conversely, FAAH activators or gene therapy approaches should enhance AEA clearance in disorders characterized by FAAH deficiency, such as PCOS or POI [13,21].

FAAH-targeted interventions may protect ovarian reserve and delay age-related functional decline (Figure 12). In experimental models, FAAH activity correlates with granulosa cell survival, oocyte quality, and luteal integrity [75,78,80,88]. By preserving AEA homeostasis, such interventions can mitigate oxidative stress, inflammatory signaling, and apoptosis within ovarian cells, potentially extending reproductive lifespan [75,96].

Combining FAAH modulation with standard hormonal therapies may enhance treatment outcomes (Figure 12) [5,8,16]. For example, restoring FAAH activity alongside controlled gonadotropin stimulation could optimize follicular development and steroidogenesis, while maintaining appropriate AEA levels to support oocyte competence and luteal function [12].

Therapeutic targeting of FAAH requires precise control of enzyme activity to avoid systemic ECS disruption (Figure 12) [28,91,92,93,94,95,96,97,98,99,100,101]. Tissue selectivity, dose optimization, and long-term safety must be carefully evaluated [28]. Additionally, the interplay between FAAH, microRNAs, and epigenetic regulators suggests that combined molecular approaches may be necessary to achieve robust and sustained ovarian microenvironment modulation [38,39,40,41,42].

Further studies should elucidate the mechanisms governing FAAH expression and activity in ovarian cell subtypes and across reproductive phases. Accordingly, systemic FAAH inhibition is unlikely to be broadly beneficial for ovarian function, and FAAH inhibition should be reframed as context-dependent, largely hypothetical in ovarian disorders, potentially applicable only under highly specific, localized, or temporally controlled conditions. Therefore, further understanding how hormonal, paracrine, and environmental signals converge on FAAH will inform the design of targeted therapies that restore local AEA homeostasis, enhance follicular and luteal function, and improve reproductive outcomes.

## 7. Conclusions

FAAH is a central regulator of the ovarian microenvironment, controlling local AEA concentrations to coordinate granulosa cell survival, oocyte maturation, steroidogenesis, ovulation, and luteal function. Its cell-specific expression and dynamic regulation by hormones, microRNAs, and epigenetic mechanisms ensure proper intraovarian signaling and reproductive competence.

Dysregulation of FAAH may disrupt ovarian AEA homeostasis, impairing follicular development, luteal maintenance, and endometrial receptivity, and contribute to reproductive disorders such as PCOS, POI, and infertility. Mechanistic evidence highlights that FAAH deficiency perturbs paracrine interactions between granulosa, theca, and luteal cells, emphasizing its role in maintaining a balanced ovarian microenvironment.

Targeting FAAH may represent a potential therapeutic strategy to restore intraovarian AEA balance, preserve ovarian reserve, and improve fertility outcomes. Future research should focus on clarifying FAAH regulation within specific ovarian cell types, the integration of hormonal and paracrine signals, and the development of safe, tissue-selective modulators to manipulate FAAH activity.

Overall, understanding the molecular mechanisms of FAAH in ovarian physiology and pathology may provide critical insights into reproductive biology and offer avenues for novel interventions aimed at optimizing female fertility and delaying ovarian aging.

## Figures and Tables

**Table 1:** Fatty acid amide hydrolase (FAAH) expression and mechanistic regulation in ovarian tissues across different species.

Species	Experimental Model	Ovarian Cell Type/Tissue	Key Findings Related to FAAH	Reference
Mouse	In vivo, uterus/ovary	Whole ovary, uterine tissue	FAAH expression is downregulated by estrogen and progesterone, leading to increased anandamide levels during the peri-implantation period	MacCarrone et al., 2000 [17]
Mouse	In vitro	Granulosa cells	FAAH activity modulates anandamide tone, influencing follicular development and steroidogenic balance	Wang et al., 2006 [45]
Rat	In vivo	Ovary	Endocannabinoid signaling components, including FAAH, are dynamically regulated during the estrous cycle	Battista et al., 2012 [46]; Walker et al., 2019 [47]
Bovine	In vitro	Granulosa and cumulus cells	FAAH expression correlates with follicular size and oocyte competence	Cecconi et al., 2020 [48]
Mouse	Knockout model	Whole ovary	Genetic or pharmacological suppression of FAAH alters ovulation efficiency and follicular dynamics	Rossi et al., 2007 [29]; Wang et al., 2006 [45]
Human	Ex vivo	Granulosa/luteal cells	FAAH expressed and regulates intraovarian AEA homeostasis	Taylor et al., 2010 [49]

**Table 2:** Fatty acid amide hydrolase (FAAH) and endocannabinoid signaling in human ovarian physiology and disorders.

Species	Sample Type	Ovarian Condition	FAAH-Related Findings	Reference
Human	Ovarian tissue	Normal ovary	FAAH is expressed in granulosa and luteal cells, contributing to local endocannabinoid homeostasis	Taylor et al., 2010 [49]
Human	Endometrial—ovarian interface	Infertility	Reduced FAAH expression is associated with elevated anandamide levels and impaired reproductive outcomes	Maccarrone et al., 2001 [87]
Human	Clinical samples	PCOS	Dysregulation of FAAH and ECS components may contribute to altered steroidogenesis and follicular arrest	El-Talatini et al., 2009 [16]
Human	Peripheral blood/ovarian tissue	Infertility	Lower FAAH activity correlates with adverse reproductive outcomes	Trabucco et al., 2009 [88]
Human	Review synthesis	Ovarian disorders	FAAH emerges as a potential therapeutic target in ovarian dysfunction	Battista et al., 2012 [46]; Walker et al., 2019 [47]

## Data Availability

No new data were created or analyzed in this study. Data sharing is not applicable to this article.

## Abbreviations

The following abbreviations are used in this manuscript:

AEA	anandamide
Akt/PKB	protein kinase B
BMP15	bone morphogenetic protein 15
CB1	cannabinoid receptor 1
CB2	cannabinoid receptor 2
CYP17A1	cytochrome P450 family 17 subfamily A member 1
CYP19	CYP19A1, ytochrome P450 family 19 subfamily A member 1
ECS	endocannabinoid system
ER	endoplasmic reticulum
FAAH	fatty acid amide hydrolase
GDF9	growth differentiation factor 9
GnRH	gonadotropin-releasing hormone
H3K9ac	histone H3 acetyl Lys9
IL-6	interleukin-6
LIF	leukemia inhibitory factor
MAPK	mitogen-activated protein kinase
OSE	ovarian surface epithelium
Pappa	pregnancy-associated plasma protein-A
PCOS	polycystic ovary syndrome
PI-3K	phosphatidylinositol 3-kinase
POI	premature ovarian insufficiency
Ptgs2	cyclooxygenase-2
StAR	steroidogenic acute regulatory protein
TNF-α	tumor necrosis factor-α
